# Supplementary material for: Different brain systems support learning from received and avoided pain during human pain-avoidance learning
Source: eLife. 2022 Jun 22;11:e74149. doi: 10.7554/eLife.74149 (PMC9217130; doi:10.7554/eLife.74149)
Supplement: Figure 2—source data 1. [file elife-74149-fig2-data1.docx]

**Figure 2 – Source data 1.** 95% highest density intervals of the posterior distributions shown in Figure 2A.

|  | placebo | levodopa | naltrexone |
| --- | --- | --- | --- |
| $\bar{\alpha}_{pain}$ | 0.53-0.90 | 0.47-0.85 | 0.55-0.87 |
| $\bar{\alpha}_{no-pain}$ | 0.20-0.47 | 0.49-0.84 | 0.58-0.94 |
| $\bar{\beta}$ | 6.3-11.7 | 3.9-7.2 | 4.2-7.3 |
